# Assessment of Upper Limb Nerves in Coronary Artery Disease Patients Undergoing Coronary Artery Bypass Graft

**DOI:** 10.7759/cureus.66598

**Published:** 2024-08-10

**Authors:** Hitha Antony, Sunil Chouhan, Santosh Wakode, Ruchi Singh, Yogesh Niwariya, Danish Javed

**Affiliations:** 1 Physiology, All India Institute of Medical Sciences, Bhopal, Bhopal, IND; 2 Cardiothoracic and Vascular Surgery, All India Institute of Medical Sciences, Bhopal, Bhopal, IND; 3 AYUSH (Ayurveda, Yoga and Naturopathy, Unani, Siddha, Sowa Rigpa, and Homoeopathy), All India Institute of Medical Sciences, Bhopal, Bhopal, IND

**Keywords:** recovery, nerve damage, surgical effects, postoperative complications, open heart surgery

## Abstract

Background

Many patients experience pain in their upper limbs following surgical procedures involving median sternotomy, particularly those undergoing coronary artery bypass grafting (CABG). This type of pain, commonly reported by CABG patients, is often overlooked in hospital settings. Our study aims to address this issue by utilizing electrodiagnostic studies to understand this postoperative discomfort better.

Objectives

Cardiovascular procedures are standard and are trending toward endovascular interventions. Through this study, we aim to assess the occurrence of neurological issues in the upper limbs after CABG by comparing patients' preoperative and postoperative electrophysiological studies of the upper limb nerves.

Materials and methods

A prospective study was performed on 32 coronary artery disease (CAD) patients undergoing CABG to determine the effects of surgery on the upper limb nerves (median and ulnar nerves). We performed nerve conduction studies (NCS) and analyzed different parameters of both median and ulnar nerves pre and post-surgery.

Results

A change was noted in different NCS parameters of the median and ulnar nerves when we compared the pre and post-surgical values. The mean latency of the median nerve sensory increased from a minimum of 3.01 milliseconds at the preoperative level to a maximum of 3.60 milliseconds when assessed two weeks post-surgery. The mean amplitude decreased from 16.49 microvolts to a minimum of 12.30 microvolts when assessed two weeks post-surgery. The mean velocity decreased from 55.83 m/s at the preoperative value to a minimum of 45.03 m/s at the two weeks post-surgery assessment. The ulnar nerve also underwent similar changes.

Conclusion

The observed changes in latency, amplitude, and velocity might be attributed to various factors, including surgical trauma, inflammation, or alterations in the physiological state post-surgery. The sternotomy technique and the position and extent of opening the sternal retractor determine the prevalence of complications by causing injury to the medial and lateral cords of the brachial plexus after CABG. Careful preoperative and postoperative assessments of patients may aid in preventing, minimizing, and treating these often undiagnosed complications.

## Introduction

Coronary artery bypass grafting (CABG) surgery is the established method for re-establishing blood flow in cases of severe blockage in the left main or three coronary arteries. Access for the procedure is through a midline sternotomy, which is performed either with on-pump, where the heart is stopped and perfusion is maintained through a cardiopulmonary bypass machine, or off-pump techniques, where the heart continues to beat. Suppose the most critical and direct goal in managing patients with complex coronary artery disease (CAD) is reducing cardiac death or myocardial infarction (MI)-related death. In that case, CABG surgery appears to be the preferred revascularization approach to achieve these objectives. Research indicates that individuals with numerous concurrent medical conditions and severe cardiac disease have better outcomes with surgery compared to maximal medical management. Patients undergoing cardiac or vascular surgeries have a high risk for various perioperative complications, in part due to coexisting comorbidities. Firm evidence exists that the techniques of incision and retraction of the sternum are crucial in the pathogenesis of injury to the medial and lateral cords of the brachial plexus after cardiac operations [1,2].

Upper limb neuropathies are common complications encountered during the CABG procedure [3]. Following the operation, numbness in the left forearm and hand, particularly in the medial aspect, was commonly seen as soon as the patient had awakened from anesthesia. Many factors have contributed to this. Approximately 30% of patients develop chronic poststernotomy pain (CPSP) following cardiac surgery with sternal retraction, where the sternum can be opened over 15/30 minutes [4]. Injury to the nerves of the brachial plexus after surgery is frequently observed due to the stretching of the brachial plexus during sternotomy and the application of sternal retractors in open-heart procedures [5]. The most common cause of injury is the superior rotation of the first rib and subsequent downward displacement of the clavicle, thereby stretching and injuring the brachial plexus, most often the lower roots, C8-T1 [6]. During CABG, the clavicle is pulled inferiorly as the patient's arms are pulled down and fixed at the sides. The often-used interscapular rolled towel contributes to this by moving the clavicles dorsally. The brachial plexus is susceptible to injury because it is superficial, its roots are fixed proximally and distally (making it susceptible to stretch injury), and its trunks pass through the narrow space between the first rib and clavicle. Factors that lead to nerve injury have been listed as depression of the clavicle into the sternoclavicular space, compression of the plexus between the clavicle and the first rib, lateral deviation and extension of the patient's head, stretching of the plexus over the humeral head with the arm in external rotation and extension [7]. The study aims to evaluate the changes in upper limb nerves pre and post-CABG and emphasize the importance of the nerve conduction study (NCS) in establishing the early diagnosis of postoperative nerve changes after a major operation.

## Materials and methods

Study design

This was an observational longitudinal study.

Study setting

This was a hospital-based study.

Study population

The study population consisted of CAD patients.

Study participants

We enrolled 32 CAD patients who were posted for elected CABG and consented to participate. We calculated the sample size using G*Power software. A total of 25 subjects are needed to assess the difference in the different parameters of patients before and after CABG as per the effect size achieved in previous related studies. Thus, in the present study, we have enrolled 32 participants, considering a dropout of 20%. The effect size from the reference study was utilized to determine our sample size [8]; the effect size f = 0.7624929.

We conducted the study in the Department of Physiology in collaboration with the Department of Cardiothoracic Vascular Surgery (CTVS) at AIIMS, Bhopal. The diagnosis of CAD in patients undergoing CABG was done by invasive coronary angiography in the department of CTVS. All the CAD patients who were diagnosed with double or triple vessel disease were posted for elective CABG, and those sets of patients were included in our study. The patient underwent preoperative routine laboratory investigations of Hb, platelet count, WBC count, urea, creatinine, thyroid profile, and liver function tests. If the tests were standard, which means that if all the investigations were in the normal range, patients were assigned a date for surgery and sent for a pre-anesthetic check-up and further steps for surgery. We obtained informed consent from the participants.

Then, we took them to the Physiology department for preoperative assessment. History-taking and examination were done to exclude any neurological abnormalities. Afterward, we conducted a preoperative NCS of the two upper limb nerves (median and ulnar) for the baseline records. Preoperative assessments were done after patients were admitted to the CTVS ward for their CABG procedure. One week after the CABG procedure, the patients had been discharged. An additional week later, during their first review with the CTVS surgeon, patients were again transferred to our department's neurophysiology lab, and we performed the postoperative assessment of the NCS of the above-mentioned upper limb nerves. The pre and postoperative assessments of the median and ulnar nerves were then compared and analyzed to assess the surgery's effect on different NCS parameters. We have carried out the motor nerve conduction study and sensory NCS of these nerves using standard procedure, taking all the needed precautions and recording all the relevant parameters.

We have enrolled a total of 32 participants in our study. After completing their baseline assessment, four patients expired during their postoperative period so they couldn’t be included in the postoperative study. Hence, we did not include them in our data analysis. We have thus analyzed the data(both pre and post) of the remaining 28 patients and the results are as follows.

In all our CABG patients, a great saphenous vein graft (GSV) was used, which was harvested from the leg. All the patients were administered general anesthesia. This typically involved the induction of anesthesia using intravenous agents, followed by maintenance with inhalational anesthetics and muscle relaxants as required. The anesthetic management was closely monitored throughout the surgery to ensure optimal conditions for the procedure. Hemodynamic monitoring was done using invasive arterial lines and central venous catheters. As the GSV was used in all our patients, harvested from the leg, vascular access in the lower limb was avoided. An invasive arterial catheter was placed typically in the radial artery for continuous blood pressure monitoring and arterial blood gas sampling. A central venous catheter was inserted in the internal jugular vein to administer medications and fluids and monitor central venous pressure. Non-invasive blood pressure (NIBP) monitoring with an appropriately sized cuff was placed on the upper arm. Upper limb vascular access was prioritized to prevent complications and to preserve the lower limb integrity of vein harvesting. Peripheral nerve block, like intercostal nerve block, was used to relieve pain by targeting specific intercostal nerves.

Exclusion criteria

Patients who did not provide consent, patients requiring emergency CABG, and patients prescribed neurotoxic medications like Ethambutol, Isoniazid, and Cetuximab were not eligible for inclusion in the study. Patients undergoing emergency CABG were excluded from our analysis due to their hemodynamic instability, which made preoperative assessment impractical. We focused exclusively on elective CABG patients who were clinically stable. Additionally, to ensure the accuracy of our NCV measurements, we specifically screened for medications known to affect NCV such as Ethambutol, Isoniazid, and Cetuximab. Only patients not taking these medications were included in the study.

Ethical considerations

The study commenced following approval from the Institutional Human Ethics Committee of AIIMS, Bhopal (Ethics Permission No: IHEC-PGR/2022/PG/Jan/33, dated September 12, 2022). We obtained written consent from the patients before enrollment in the study.

Study period

The study lasted for one and a half years, from November 2022 to January 2024, with data analysis taking two months.

Nerve conduction study (NCS)

The NCS is a valid electrodiagnostic tool routinely used in neurophysiology clinics, which can be used as a diagnostic tool to assess nerves, determine patients' prognoses, and predict recovery outcomes in a wide variety of patients. We have used it in patients undergoing CABG where postoperative neurological complications are highly likely to occur. In the present study, we employed NCS to evaluate the median and ulnar nerves and to compare values of the pre and post-CABG assessments of the neurophysiological parameters of these nerves. Various parameters recorded during a sensory NCS were latency, sensory nerve action potential (SNAP) amplitudes, and conduction velocity (CV). On the other hand, motor NCS involves documenting onset latencies, compound muscle action potential (CMAP) amplitudes, and NCV. We have used the Nihon Kohden Neuropack X1 MEB 2300 machine (Irvine, CA, USA) available in the neurophysiology lab at our institution to conduct nerve conduction studies for our research. The nerves tested in my research were the median and ulnar nerve. We have analyzed the motor and sensory parts of each nerve. We have carried out only a one-sided assessment and comparison.

Our statistical analysis was carried out using IBM SPSS statistics version 28 (IBM Corp., Armonk, NY, USA). We have used the non-parametric statistical tests due to the data's non-normal distribution. Specifically, we utilized the paired Wilcoxon test to examine changes in various median and ulnar nerve sensory parameters in the NCS between the two time points (pre and post-CABG).

## Results

When we consider the demographic details of participants, we have found that the mean age in years was 57.38 ± 7.88, the mean height in cm was 164.92 ± 6.92, and the mean weight in kg was 66.31 ± 8.3. Considering the age, the three criteria (skewness, kurtosis, Shapiro-Wilk test), only one of the three criteria was suggestive of normality; it appeared that the data was not normally distributed (did not follow a bell-shaped curve). Considering the same criteria in height, the data was normally distributed since all three criteria (skewness, kurtosis, Shapiro-Wilk test) were suggestive of normality. Considering the weight, data appeared normally distributed since all three criteria suggest normality. Table 1 depicts the demographic features of the patient in terms of mean age, height, and weight.

**Table 1:** Distribution of participants based on their demographic details

Parameter	Mean SD	P value
Age (years)	57.38 (7.88)	0.016
Height (cm)	164.92 (6.92)	0.270
Weight (kg)	66.31 (8.30)	0.465

 We have conducted NCS on one side and then compared the pre and post-surgical values of NCS. We have employed the non-parametric statistical tests due to the non-normal distribution of the data. The paired Wilcoxon test was utilized to investigate the variation in the different parameters of the NCS of the median and ulnar nerve sensory between the two time points (pre and post-CABG). Table 2 depicts the changes in different NCS parameters of the median nerve sensory.

**Table 2:** Changes in different parameters of NCS of the median nerve sensory NCS: nerve conduction studies

Timepoint	Different parameters of NCS of the median nerve sensory		
	Latency in milliseconds (SD)	Amplitude in microvolts (SD)	Velocity in m/s(SD)
Preoperative	3.01 (1.41)	16.49 (5.53)	55.83 (11.73)
2 Weeks	3.60 (1.55)	12.30 (6.56)	45.03 (11.81)
Absolute Change	0.59 (1.74)	4.18 (6.18)	10.80 (12.11)
Percent Change	29.2% (53.0)	20.8% (40.7)	16.5% (27.2)
P value	0.021	0.003	<0.001

The mean latency of the median nerve sensory increased from a minimum of 3.01 milliseconds at the preoperative level to a maximum of 3.60 milliseconds when assessed two weeks post-surgery. This was a statistically significant change (Wilcoxon test: V = 69.0, p = 0.021). The mean amplitude of the median nerve sensory decreased from 16.49 microvolts at the preoperative time to a minimum of 12.30 microvolts when assessed two weeks post-surgery, a statistically significant change. (Wilcoxon test: V = 290.0, p = 0.003). The mean velocity of the median nerve sensory of the selected side decreased from 55.83 m/s at the preoperative value to a minimum of 45.03m/s at the two weeks post-surgery. This change was also statistically significant (Wilcoxon test: V = 330.0, p = <0.001).

We have analyzed the ulnar nerve, too. Similar changes were observed in various parameters of the ulnar nerve as well. Table 3 depicts the changes in different NCS parameters of the ulnar nerve sensory.

**Table 3:** Different parameters of NCS of the ulnar nerve sensory of the selected side over time NCS: nerve conduction studies

Timepoint	Different parameters of NCS of the ulnar nerve sensory of the selected side		
	Latency in milliseconds (SD)	Amplitude in microvolts (SD)	Velocity in m/s(SD)
Preoperative	2.60 (1.31)	13.69 (6.53)	56.17 (12.69)
2 Weeks	4.98 (4.64)	10.50 (6.11)	39.91 (13.20)
Absolute Change	2.38 (4.56)	3.18 (5.36)	16.26 (12.90)
Percent Change	19.1% (194.9)	0.1% (87.7)	28.1% (19.3)
P value	<0.001	0.004	<0.001

The mean latency of the sensory component of the ulnar nerve has increased from a minimum value of 2.60 milliseconds during the preoperative recording to a maximum of 4.98 milliseconds at the two weeks post-CABG recording, and this change was statistically significant (Wilcoxon test: V = 26.5, p = <0.001). The mean amplitude of the ulnar nerve sensory decreased from a preoperative value of 13.69 microvolts to 10.50 microvolts at the two weeks post-CABG recording. The mean conduction velocity of the ulnar sensory nerve showed a decrease from a maximum of 56.17 m/s at the preoperative value to a minimum of 39.91 m/s at the two-week postsurgical value, which was a statistically significant change (paired t-test: t = 6.4, p = <0.001).

## Discussion

The NCS is a valid electrodiagnostic tool that objectively determines the functions of the peripheral nerves. In our present study, we employed NCS to evaluate the sensory component of the median and ulnar nerves. We found that the comparative values of the pre and post-CABG were different in both the tested upper limb nerves. A meaningful shift was observed in various parameters of the sensory nerves during the two-week postoperative period. When we assessed both nerves, similar findings were observed. Latency prolongation, a decrease in conduction velocity, and a reduction in sensory nerve action potential amplitude were observed. An increase or prolongation in the latency suggests a delay in transmitting sensory signals along the nerve, indicating a possible impact on nerve function or integrity. The sensory nerve action potential's amplitude decreased, which reflects a decrease in the magnitude of the nerve's electrical response, which could signify a compromise in the integrity or function of the median nerve. A reduction in conduction velocity indicates a slowing down of the conduction speed of the nerve along the sensory pathway. The statistical significance of these changes emphasizes its clinical relevance and suggests a considerable impact of surgery on the tested nerves during the two-week postoperative assessment. The reasons for the observed changes were various factors, including surgical trauma, inflammation, or alterations in the physiological state post-surgery. The statistical significance, coupled with the magnitude of the observed changes, underscores the importance of closely monitoring neurophysiological parameters in the postoperative period.

In a study conducted by Kirsh et al., in five cases treated at the University of Michigan Hospital, different levels of paralysis in the upper limbs occurred due to excessive widening of the retractors used to enhance heart exposure in heart surgery. There were varying degrees of motor or sensory deficit in the upper extremities and shoulder girdles. Patients developed partial or complete paralysis as soon as they awoke following anesthesia. In all patients, the function gradually returned to normal over several months. While malpositioning the unconscious patient, which may happen when the operation requires the Trendelenburg position or during the hyperabduction of the arm, abnormal tension, and stretch forces are generated.

In the prospective study conducted by Seyfer et al., in 53 patients who had cardiac surgery that used the standard median sternotomy, detailed sensory and motor assessments were conducted, and measurements of sternal retraction distance and cardiopulmonary bypass duration were recorded during surgery. Twenty patients (37.7%) developed motor and sensory neuropathies postoperatively, all of which affected the ulnar nerve. Five patients who underwent electromyography and nerve conduction assessments showed signs of brachial plexus injury. On average, the duration of symptoms was 2.3 months, but some patients continue to experience unresolved symptoms in the long term [9]. A lesion involving the brachial plexus may represent neurapraxia (possibly a compression injury of the medial cord between the soft tissues about the clavicle and the first rib during sternal retraction).

Because of its dorsal articulations, the first rib can only rotate upward during retraction. The typical patient positioning also contributes to the arms being pulled downward. When the sternum is later split and retracted, the first rib is forced upward, thereby infringing on the cone through which the plexus exits. Ulnar neuropathies are common after cardiac surgery. However, they are often not detected in the initial postoperative phase since patients may not be conscious of symptoms associated with the condition. NCS is, therefore, helpful in the early detection of neuropathy and localizing the lesion.

Patients who received coronary artery bypass graft surgery experienced peripheral nervous system (PNS) complications postoperatively. The most common is brachial radiculoplexopathy, which occurs in most patients and involves the lower trunk or medial cord fibers. A relationship was observed between the location of jugular vein cannulation and the affected side, indicating potential involvement of needle trauma. Some cases may have been influenced by stretching resulting from chest wall retraction. Other deficits included saphenous, common peroneal, and phrenic nerve engagement, singular vocal cord paralysis, and partial Horner's syndrome were observed. A higher likelihood of PNS complications was noted in males. Elevated risk was linked to hypothermia experienced during surgery [10]. Other nerves were also involved. Nerve conduction studies and needle electromyography indicated a partial conduction block of the radial nerve within the spiral groove. And motor axonal loss distal to the site of the lesion. Extended periods of patients lying on the operating table may have led to compression of their left upper arm against the supporting column of the self-retractor, fixed to the table rail 5 cm above the left elbow joint, in the site where the radial nerve is directly opposed to the humerus [11]. Many patients suffer from another frequent complication which is post-CABG pain (PCP) syndrome, which is a chronic, unusual pain attributed to a range of factors such as brachial plexus traction, scar discomfort, costochondral junction pain, and complex regional pain syndrome affecting the upper limb. Here, the patient complained of left upper back pain on the thoracic first to fourth dermatomes, numbness, and a cold sensation in the left arm after CABG [12].

Inflammatory etiologies are also becoming increasingly recognized as explanations of nerve changes, especially those occurring in the perioperative period [13]. When we think about what we can do to prevent these, there are certain practices to reduce or prevent sternal complications. It involves the routine prescription of sternal precautions immediately after surgery. These precautions limit the immediate use of the upper limbs for 6 to 12 weeks following surgery. Patients are encouraged not to use their upper limbs during everyday tasks such as bed transfers or lifting objects. The rationale for these restrictions is to promote solid osteosynthesis and bone healing by minimizing the forces and the amount of micromotion between the sternal edges, which can encourage progression to non-union and infection [14]. However, the most critical early measures are careful sternal retraction and using the hands-up position for low incidence and benign course of nerve injury problems [15]. The hands-up position not only appears to prevent upper limb nerve plexus injury from the posterior displacement of the shoulder but may minimize compression injury by first rib rotation during retraction of median sternotomy. This position also benefits the anesthesiologist in providing direct, immediate access to intravenous and intra-arterial cannulas [16]. The high incidence of conduction slowing across the nerves suggests that electrophysiological studies are critical for accurately assessing the actual frequency of postoperative occurrences, such as neuropathies [17]. There's considerable diversity in how hospitals approach sternal precautions and protocols for patients post-median sternotomy [18]. Patients would benefit from a less stringent and more engaged care plan, which is advised for recovering from cardiac surgery requiring median sternotomy [19].

Recommendations to reduce the risk of postoperative ulnar neuropathy include anatomically neutral arm positioning and padding intraoperatively [20]. When we consider brachial plexopathies, 10% are iatrogenic, most commonly caused by stretching or compression of nerve tissue during procedure-related positioning such as in CABG [21].

Our present study had certain limitations, CABG procedures are performed less frequently compared to other regions, which limited the pool of potential participants. Ours was a single-center study, as it was conducted at a single institution, which may limit the generalizability of the findings to other settings or populations. In all our CABG patients, a great saphenous vein graft (GSV) was used, which was harvested from the leg. Hence, vascular access in the lower limb was avoided. Upper limb vascular access was prioritized for hemodynamic monitoring using invasive arterial lines, a central venous catheter, and NIBP monitoring. It is unclear about NCS deficits that might have been raised from such procedures. This topic needs further research. Although we had assessed the patients during further reviews, the lack of follow-up of NCS to track the progression of these symptoms experienced by patients limits our ability to draw definitive conclusions about the development of chronic postoperative pain.

## Conclusions

It is recognized that heart surgery can occasionally result in upper limb nerve injuries. These issues are often not deemed clinically significant and thus aren't typically investigated. It's essential to closely monitor patients undergoing open heart surgery for potential peripheral nerve injuries during the postoperative period. Our study concludes that cardiac surgery significantly impacts peripheral nerves and may lead to gradually progressing neuropathy. Consequently, it is recommended to implement close follow-up, management strategies, and standardized electrodiagnostic guidelines for early diagnosis of neural function to ensure optimal recovery. The above observations highlight NCS's importance in establishing early diagnosis of postoperative nerve changes after a major operation. This study also paves the way for more benefits to similar subsets of patients in the future.

Clinical practice recommendations include performing a comprehensive NCS preoperatively on patients scheduled for CABG to establish a baseline of nerve function, conduction of NCS at regular intervals post-surgery to monitor any changes in nerve function, use of NCS results to identify early signs of nerve injury or dysfunction, allowing for timely interventions such as physical therapy or medications to mitigate nerve damage and promote recovery, and multidisciplinary approaches such as integration of NCS findings into the broader perioperative care plan, involving neurologists, cardiologists, and rehabilitation specialists to optimize patient outcomes. Future research recommendations include conducting longitudinal studies to track nerve conduction changes over extended periods post-CABG, helping understand the long-term impact of the surgery on nerve health. Investigate patient-specific factors (e.g., age, diabetes, comorbidities) that may influence susceptibility to nerve damage during and after CABG.
